# Dynamic Changes in Neutral and Acidic Ginsenosides with Different Cultivation Ages and Harvest Seasons: Identification of Chemical Characteristics for *Panax ginseng* Quality Control

**DOI:** 10.3390/molecules22050734

**Published:** 2017-05-04

**Authors:** Zhi Liu, Chong-Zhi Wang, Xing-You Zhu, Jin-Yi Wan, Jing Zhang, Wei Li, Chang-Chun Ruan, Chun-Su Yuan

**Affiliations:** 1 Institute of Agricultural Modernization, Jilin Agricultural University, Changchun 130118, China; lzhiiu@126.com; 2 College of Chinese Medicinal Materials, Jilin Agricultural University, Changchun 130118, China; zhjing0701@163.com; 3 Tang Center for Herbal Medicine Research and The Pritzker School of Medicine, University of Chicago, Chicago, IL 60637, USA; czwang@dacc.uchicago.edu (C.-Z.W.); wanjinyi1128@163.com (J.-Y.W.); cyuan@dacc.uchicago.edu(C.-S.Y.); 4 Jinlin Provincial Sericulture Institute, Jilin 132012, China; zhuxy1961@sina.com

**Keywords:** *Panax ginseng*, Ro/Re ratio, Rg_1_/Re ratio, malonyl ginsenoside, cultivation age, harvest season, quality control

## Abstract

In this study, dynamic changes in ginsenoside content and ratios in the *Panax ginseng* root were investigated with different cultivation ages and different collection months, using high-performance liquid chromatography (HPLC). Our data indicate that changes in ginsenoside Ro and malonyl ginsenosides content were dependent on the ginseng cultivation age (*p* < 0.05); especially, the Ro content varied from 0.16 to 4.91 mg/g, with a difference about 30-fold. Further, we found that the samples of 5 and 6-year-old *P. ginseng* had high Ro/Re ratio, whereas two and three-year-old *P. ginseng* possessed low Ro/Re ratio. Thus, the Ro/Re ratio can be used as a characteristic marker for differentiating the age of the root. The relative content of ginsenosides Rg_1_ and Re were affected by the ginseng’s harvest season. The Re content was higher than the Rg_1_ content in May and June, but lower than the Rg_1_ content from August to October. Thus, the Rg_1_/Re ratio can be used as a characteristic marker for differentiating the ginseng’s harvest seasons. These results indicate that the chemical characteristics of *P. ginseng* at different cultivation ages and harvest seasons are clearly different, which may cause differences in pharmacological activities and therapeutic effects. In addition, we developed HPLC coupled with hierarchical cluster analysis and principal component analysis methods to identify the cultivation age and harvest season of *P. ginseng* using characteristic ginsenosides. Our results showed that this method can be used to discriminate the cultivation age and harvest season of *P. ginseng.*

## 1. Introduction

**Figure 1:**
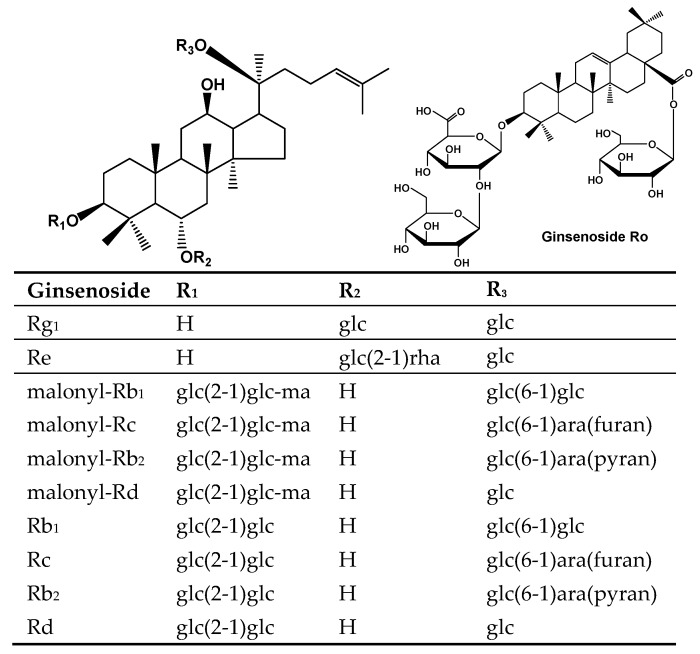
Structures of ginsenosides from *Panax ginseng* root. glc—glucosyl; ma—malonyl; ara—arabinosyl; rha—rhamnosyl.

*Panax ginseng* is a slow-growing perennial plant of the *Panax* genus that has been used in China for thousands of years. Nowadays, *P. ginseng* has become a popular dietary supplement in many countries [1]. Modern pharmacological studies have demonstrated that *P. ginseng* possesses numerous health benefits including antitumor, antioxidant, anti-obesity and antidiabetic properties [2,3,4,5,6]. The active components of ginseng are commonly considered to be ginsenosides, a group of steroidal saponins, including commonly studied neutral ginsenosides (e.g., Rg_1_, Re, Rb_1_, Rb_2_, Rc and Rd) and the less studied oleanolic acid-type ginsenosides (e.g., ginsenoside Ro) and malonyl ginsenosides (e.g., mRb_1_, mRb_2_, mRc and mRd) (Figure 1). The ginsenoside Ro and malonyl ginsenosides are also called “acidic ginsenosides”. Previous studies have shown that the content of acidic ginsenosides represents up to 60% of the total ginsenosides in Asian ginseng [7,8].

The pharmacological effects and chemical analyses of neutral ginsenosides are frequently used for the evaluation and quality control of ginseng products [9,10]. Acidic ginsenosides are more polar and water-soluble than neutral ginsenosides, and the analysis of acidic ginsenosides by HPLC is more difficult than those of neutral ginsenosides using a mobile phase without phosphate buffer [7,11,12]. In previous studies, due to the unavailability of reference standards, identification of the malonyl ginsenoside and Ro was carried out by comparing the retention times of their chromatographic peaks with published data or by high-performance liquid chromatography mass spectrometry (HPLC-MS) [1,12,13,14]. The concentrations of mRb_1_, mRb_2_, mRc, and mRd were calculated relative to each other, on the basis of the calibration curves of Rb_1_, Rb_2_, Rc and Rd respectively [12,15].

Since the levels of ginsenosides contribute to ginseng’s bioactive properties, accurately knowing the levels of ginseng saponins and their proportions is important for the pharmacological evaluation of ginseng products. It has been shown that ginseng species, population, and geographical origin are linked to different ginsenoside contents and ratios [16,17,18,19,20]. For example, Sengupta et al. [16] reported that Asian ginseng had a high Rg_1_:Rb_1_ ginsenoside ratio and Rg_1_ was shown to promote wound healing. Conversely, American ginseng had a low Rg_1_:Rb_1_ ratio and Rb_1_ was shown to inhibit tumor growth. Schlag and McIntosh [20] showed that there were different chemotypes based on Rg_1_/Re ratios in the American ginseng population (the low Rg_1_/high Re chemotype and the high Rg_1_/low Re chemotype), and Re was shown to reduce blood glucose. The heterogeneity of ginsenosides is an important issue because these structure-similar ginsenosides perform different or even totally opposite pharmacological activities.

The quality and efficacy of ginseng are closely related to its cultivation age and harvest season. Many studies have demonstrated that changing trends in the saponin contents of *P. ginseng* with the cultivation age. Shi et al. [21] reported that Re, Rc, Rb_2_ and Rb_3_ content increases with cultivation age. The Rg_1_, Rb_1_ and Rd content increases from one-year-old to four-year-old *P. ginseng*, and then decreases. Qu et al. [22] compared the major saponins in the American ginseng, and found that the Re and Rb_1_ content gradually increased from ages 1 to 5 years in the *P. ginseng* root; the same result was also observed for the changes in total saponin content. However, most previously published reports on variations with cultivation age in the content of total and individual ginsenosides of *P. ginseng* are limited to the determination of neutral ginsenosides. In addition, past studies only collected ginseng samples in fall. The accumulation characteristics of neutral and acidic ginsenosides at different cultivation ages and harvest seasons of *P. ginseng* have not yet been explored. Dynamic changes in ginsenoside content and their ratios should provide important information for accurate evaluation and quality control of *P. ginseng*.

In recent years, the adulteration of ginseng products has been a major problem in ginseng commercial markets, because high-value ginseng products are expensive, adulteration with other cheaper plant material or ginseng radix younger than five or six years old is likely to occur in the marketplace. Therefore, it is critical to establish a rapid and reliable quality control methodology to identify ginseng products. Nowadays, metabolomic approaches combined with multivariate analyses have developed into a powerful tool for comprehensively evaluating and discriminating between medicinal plants [23]. Metabolomic approaches based on various analytical techniques, including gas chromatography (GC), liquid chromatography (LC), and nuclear magnetic resonance (NMR), have been applied for metabolite profiling, in order to identify *Panax* ages [13,24,25,26]. However, very little work has been carried out on discrimination between cultivation ages and harvest seasons of *P. ginseng* root.

In the current study, dynamic changes in ginsenoside content and ratios in the *P. ginseng* root were investigated with different cultivation ages and different collection months, using HPLC. Our data indicate that changes in the contents of ginsenoside Ro and malonyl ginsenosides were clearly dependent on the ginseng’s cultivation age, and the content of ginsenosides Rg_1_ and Re were affected by the ginseng’s harvest season. The chemical characteristics for different cultivation ages and harvest seasons of *P. ginseng* are obviously different, which may cause differences in pharmacological activity and therapeutic effect.

## 2. Results

### 2.1. Method Validation

**Figure 2:**
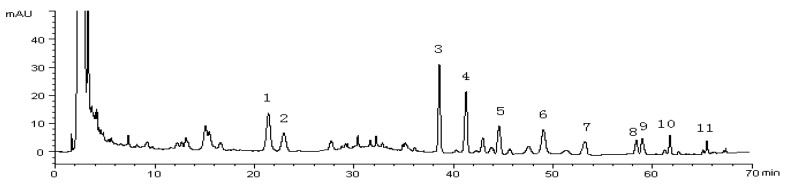
Typical HPLC chromatogram of a fresh ginseng root. Peak numbers: 1—Rg_1_; 2—Re; 3—Ro; 4—malonyl-ginsenoside Rb_1_; 5—malonyl-ginsenoside Rc; 6—malonyl-ginsenoside Rb_2_; 7—Rb_1_; 8—Rc; 9—malonyl-ginsenoside Rd; 10—Rb_2_; 11—Rd.

HPLC was used to analyze the ginseng saponins. Ginsenosides Rg_1_, Re, Ro, Rb_1_, Rc, Rb_2_, Rd, malonyl-Rb_1_, malonyl-Rc, malonyl-Rb_2_ and malonyl-Rd were identified by comparing their retention times with the mixed saponin standards. The chromatograms are shown in Figure 2. The HPLC method was validated by studying the linearity, repeatability, precision, stability, and recovery. Linearity was established by injections of the standard mixture solutions in the investigated ranges from low to high concentrations. A high degree of linearity was observed for all ginsenosides (*r*^2^ ranging between 0.9991 and 0.9999), for all standards. Repeatability was evaluated by analyzing six replicates of the same sample, and the relative standard deviation (RSD) values for the repeatability of the ginsenosides ranged from 1.96% to 3.85%. In the precision experiment, the intra-day precision was calculated six times per day and inter-day precision was analysed on 3 consecutive days, and the RSD values of both intra- and inter-day precisions did not exceed 3.61%. The stability of the sample was evaluated at the time points of 0, 2, 4, 8, 24 and 48 h with RSDs not more than 2.43%. The recoveries of ginsenosides were determined with spiked samples. Known amounts of the standard references were spiked into samples and then prepared as test solutions. The recoveries of all 11 ginsenosides were within the range of 96.9%–104.8%. The validation results demonstrated that the method was acceptable for the quantitative evaluation of ginsenosides in ginseng samples.

### 2.2. Changes of Ginsenosides in Panax ginseng at Different Cultivation Ages 

**Figure 3:**
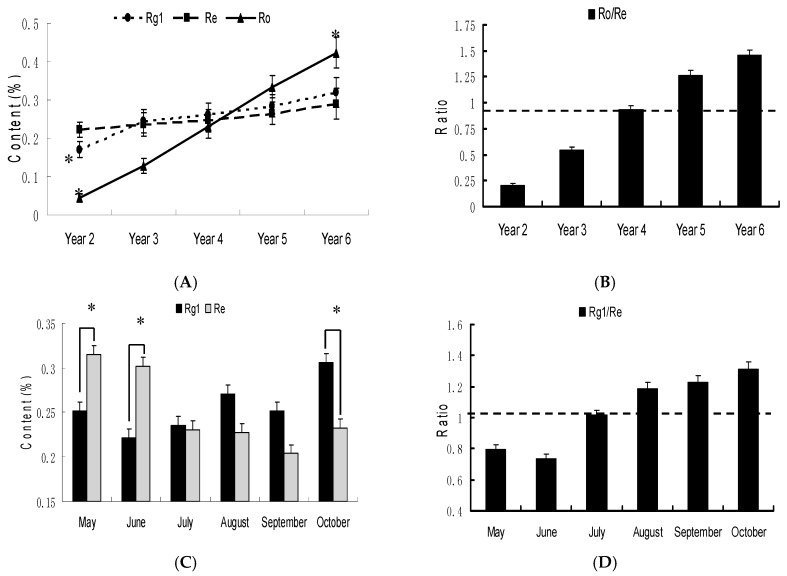
(**A**) Changes in the ginsenoside Rg_1_, Re and Ro contents in the *Panax ginseng* root according to the cultivation year. * *p* < 0.05, one-way ANOVA (compared with the Year 4); (**B**) Ratio of ginsenosides Ro and Re in different cultivation years; (**C**) Changes of ginsenoside Rg_1_ and Re contents in *Panax ginseng* root with different collection month. * *p* < 0.05, independent sample *t*-test; (**D**) Ratio of ginsenosides Rg_1_ and Re in different collection months.

Figure 3A shows variation trends in the contents of Rg_1_, Re and Ro in samples of *P. ginseng* at different ages. The average contents of Rg_1_, Re and Ro increased with cultivation age, but the rate of increase was different. In particular, the changes in Ro content are obviously dependent on age (*p* < 0.05). The average Ro content in six-year-old *P*. *ginseng* (4.228 mg/g) was approximately nine fold higher than that in two-year-old *P. ginseng* (0.445 mg/g). But, the changes in the contents of Rg_1_ and Re in root are relatively less from two to six years old, ranging from 1.705 to 3.195 mg/g and 2.22 to 2.9 mg/g, respectively.

The Ro/Re ratios were found to be different when compared across samples from two- to six-year-old *P. ginseng* (Figure 3B). The average ratio was calculated as 0.93 for four-year-old *P. ginseng*, 1.26 and 1.46 for five- and six-year-old *P. ginseng* respectively, and 0.2 and 0.54 for two- and three-year-old *P. ginseng* respectively. Our study showed that the content of Ro in four-year-old *P.* ginseng was slightly lower than that of Re, and the ratio of Ro/Re is approximately equal to 1.0. Meanwhile, the average Ro content was significantly higher than that of Re in five- and six-year-old *P. ginseng*; In contrast, the Ro content was lower than the Re content in two- and three-year-old *P. ginseng*. Therefore, the changes in the ratio of Ro/Re are obviously dependent on age. This facilitated Ro/Re ratio as a characteristic marker for differentiating the cultivation age of *P. ginseng* root.

The dynamic changes in saponin content and ratios with different cultivation ages and harvest seasons are summarized in Table 1. The Ro content in *P. ginseng* root was lowest in the May of the 2nd cultivation year and highest in the September of the 6th cultivation year, ranging from 0.16 to 4.91 mg/g, with a difference about 30-fold. The samples of five- and six-year-old *P. ginseng* had a high Ro/Re ratio, whereas two- and three-year-old *P. ginseng* possessed a low Ro/Re ratio.

**Figure 4:**
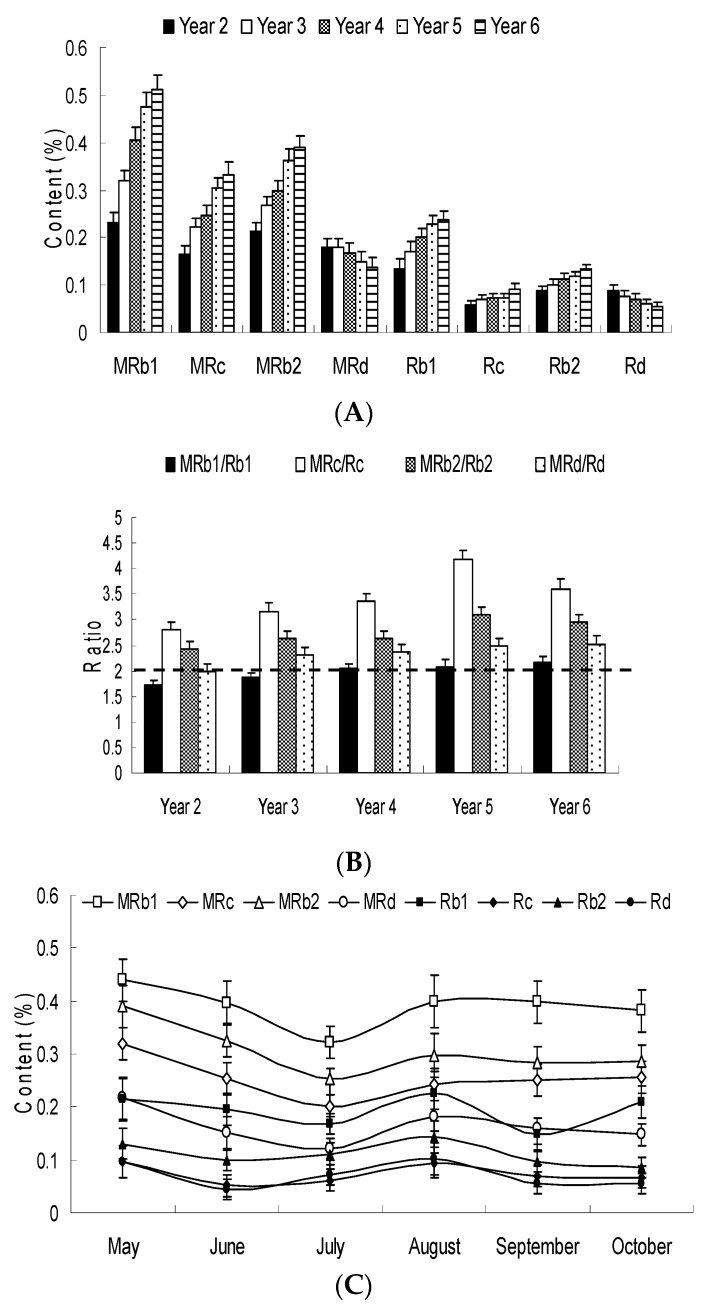
(**A**) Changes in the protopanaxadiol-type ginsenosides contents in the *Panax ginseng* root according to the cultivation year; (**B**) Ratios of malonyl ginsenosides and their corresponding neutral ginsenosides in different cultivation years; (**C**) Changes of protopanaxadiol-type ginsenosides in *Panax ginseng* root with different collection month.

As shown in Figure 4A, the average content of protopanaxadiol-type ginsenosides in *P. ginseng* root changes with age, and the change rates of the ginsenoside content were different. The changes in malonyl-Rb_1_, malonyl-Rb_2_ and malonyl-Rc content were obviously dependent on age. The Rb_1_, Rb_2_ and Rc content increased slightly with the age increase. Furthermore, the ratios of malonyl ginsenosides to their corresponding neutral ginsenosides (mRb_1_/Rb_1_, mRc/Rc, mRb_2_/Rb_2_ and mRd/Rd) ranged from 1.74 to 4.18 in different ages of *P.* ginseng root (Figure 4B). The total content of malonyl ginsenosides was two-fold higher than the content of corresponding neutral ginsenosides in fresh ginseng. These results indicate that malonyl ginsenosides are the characteristic and major constituents of fresh ginseng.

### 2.3. Changes of Ginsenosides in Panax ginseng from Different Harvest Seasons

As shown in Figure 3C, the average content of Re was significantly higher than Rg_1_ for growth in May and June in *P. ginseng* (*p* < 0.05); In July, the content of Re and Rg_1_ was nearly identical (2.36 mg/g and 2.31 mg/g, respectively), however the content of Re was lower than Rg_1_ from August to October (*p* < 0.05). Therefore, the amounts of ginsenosides Rg_1_ and Re were affected by the ginseng’s harvest season. May and June exhibited a ratio of Rg_1_/Re < 0.9 (Low Rg_1_/Re ratio), in July exhibited a ratio of 0.9 < Rg_1_/Re < 1.1 (Intermediate), and from August to October they exhibited a ratio of Rg_1_/Re > 1.1 (High Rg_1_/Re ratio) (Figure 3D). These results indicate that Rg_1_/Re ratio can be used as a characteristic marker for differentiating the cultivation month of *P. ginseng* root. Figure 4C shows changes of protopanaxadiol-type ginsenosides in *Panax ginseng* root with different collection month. The average concentrations of protopanaxadiol-type ginsenosides (mRb_1_, mRc, mRb_2_, mRd, Rb_1_, Rc, Rb_2_ and Rd) were higher in April, then decreased to the lowest level in July, then increased again during August to September, and then slightly declined in October.

### 2.4. Hierarchical Cluster Analysis (HCA) Data 

**Figure 5:**
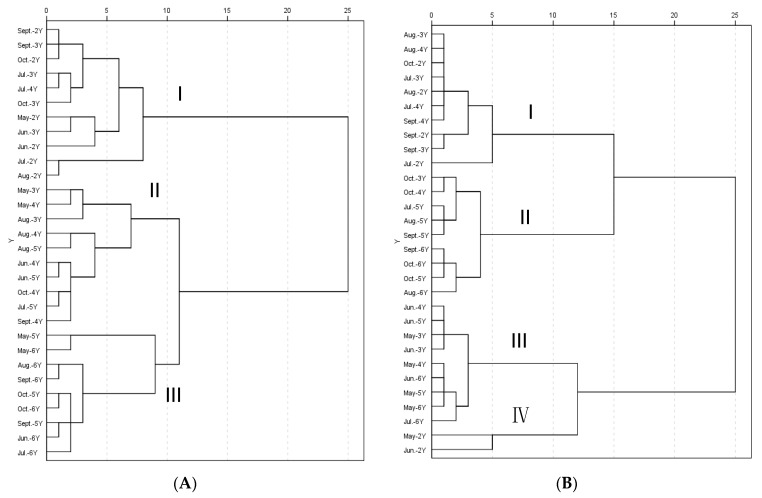
Dendrograms of hierarchical cluster analysis (HCA) using Within-groups linkage. (**A**) HCA was generated from the contents of 11 saponins in the tested samples; (**B**) HCA was generated from the contents of ginsenosides Rg_1_ and Re in the tested samples.

Hierarchical cluster analysis (HCA) was performed using the Within-groups linkage’ method, and a dendrogram was generated from the contents of 11 saponins in the *P. ginseng* samples. As shown in Figure 5A, the thirty samples were divided into three main clusters (I, II and III). Samples of two- and three-year-old *P. ginseng* were classified as cluster I. Cluster III was formed by the majority of five- and six-year-old *P. ginseng* samples and the other samples were in cluster II. Several samples including May-3Y, August-3Y, June-5Y, July-5Y and August-5Y were mixed with cluster II. In our study, the samples of *P. ginseng* were collected from plants of different ages from May to October. It was easy to obtain similar chemical qualities among close collection time the samples. Moreover, the samples of five- and six-year-old *P. ginseng* progressively merged with those of four-year-old *P. ginseng* to form a bigger cluster that finally merged with the cluster of samples of two- and three-year-old *P. ginseng* to create a whole tree structure. The result indicates that the cultivation age of *P. ginseng* root from two to six years can be discriminated based on their detected metabolites, whereas the sample’s month cannot be discriminated in this manner.

Secondly, a dendrogram of the HCA was generated using the contents of Rg_1_ and Re, the results of which are shown in Figure 5B. Ginseng samples were obviously classified into four main clusters: the *P. ginseng* from July to October contained cluster I (two- to four-year-old ginsengs) and II (five- and six-year-old ginsengs) and the *P. ginseng* from May to June included in cluster III (three- to six-year-old ginsengs) and cluster IV (two-year-old ginseng). The four ginseng clusters showed discrimination between samples at different months and ages.

### 2.5. Principal Component Analysis (PCA) Data

**Figure 6:**
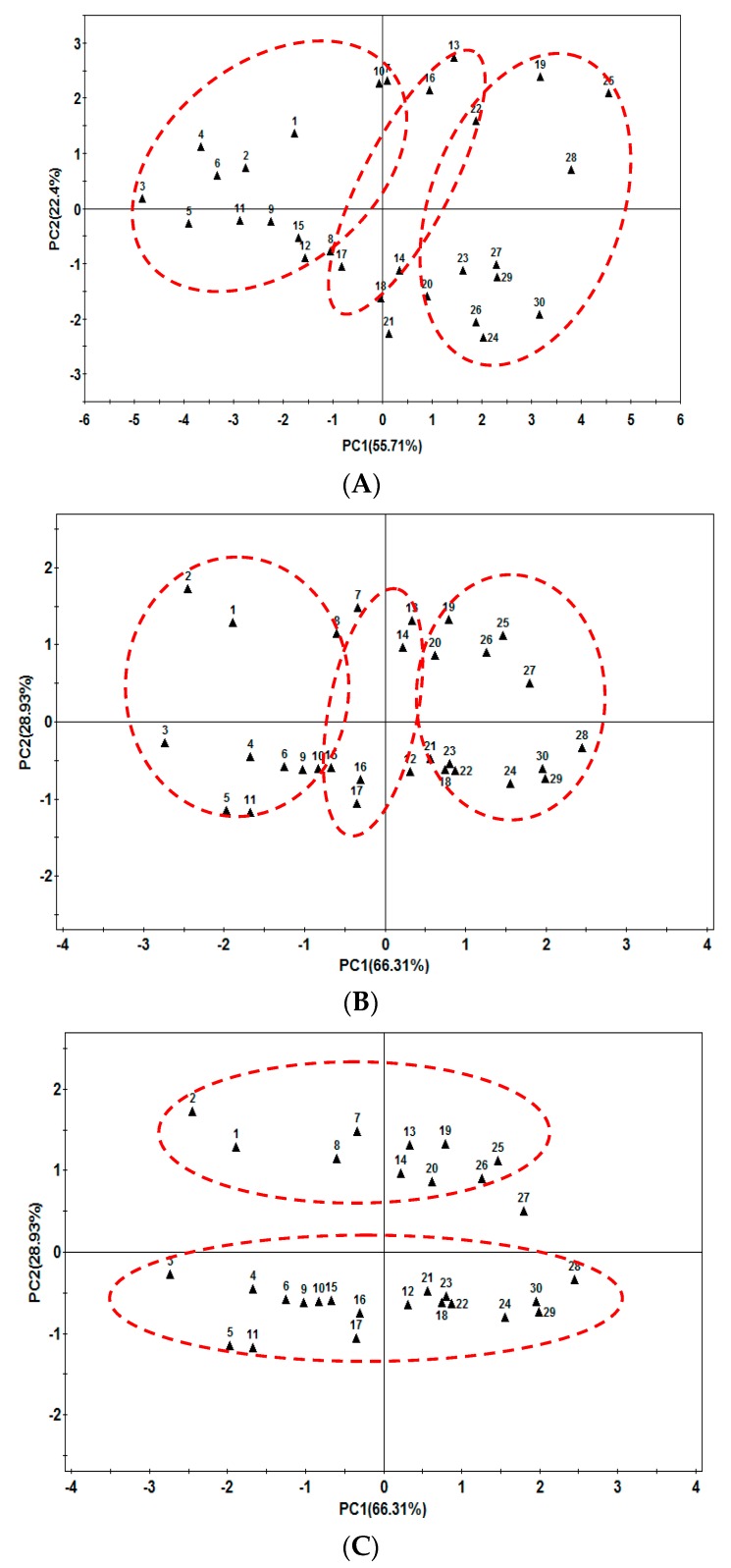
HPLC-principal component analysis (PCA) of *Panax ginseng* root. (**A**) Two-dimensional diagrams of PCA1 and PCA2, using contents of 11 saponins as input data; (**B**,**C**) Two-dimensional diagrams of PCA1 and PCA2, using contents of ginsenosides Ro, Rg_1_ and Re as input data.

Principal Component Analysis (PCA) assay was carried out on the data set obtained from the HPLC chromatogram. Eleven characteristic saponins, ginsenoside Rg_1_, Re, Ro, Rb_1_, Rb_2_, Rc, Rd, mRb_1_, mRb_2_, mRc and mRd, were chosen to build up a 11-dimensional data set, which represents 11 vectors; the contents are the value of the vectors. As shown in Figure 6A, the two-dimensional PCA score plot showed that the samples could be divided into three groups with *P. ginseng* age. Samples 1–12 (two- and three-year-old *P. ginseng*) were in group I, samples 19, 20, and 22–30 (most of the five- and six-year-old *P. ginseng*) were classified in group III, while the others were in group II.

In the case of the 30 ginseng samples, although 11 saponins were tested, most were influential in age discrimination of *P. ginseng*, and only some affected classification of the month of harvest. Thus, the sample’s month cannot be discriminated using this method. To improve the accuracy of age and month discrimination, array optimizations were performed. In this study, several relevant ginsenosides for discriminating sample age were eliminated from the array to make a smaller one. A subset of three saponins (ginsenosides Rg_1_, Re and Ro) was chosen to build the PCA model. The two-dimensional PCA score plot with the three selected saponins showed better clustering than those with all of the saponins (Figure 6B). For the two-dimensional PCA score plot, the samples collected from May to June were located in the upper area, except sample 27. In contrast, the samples from July to October were in lower area (Figure 6C).

## 3. Discussion

The pharmacological effects and chemical analyses of neutral ginsenosides (Rg_1_, Re, Rb_1_, Rb_2_, Rc and Rd) are frequently used for the evaluation and quality control of ginseng products [9,10]. But ginsenoside Ro and malonyl ginsenosides (mRb_1_, mRb_2_, mRc and mRd) have been studied less often. The activity of ginsenoside Ro is known to include anti-inflammation, antihepatitic, antioxidant and hair regrowth-promoting activities [27,28]. Malonyl ginsenosides can alleviate hyperglycemia, hyperlipemia and insulin resistance of type 2 diabetes [4]. The ginsenoside Ro and malonyl ginsenosides are abundant in Asian ginseng. The analysis of ginsenoside Ro and malonyl ginsenosides by HPLC is more difficult than those of neutral ginsenosides using a mobile phase without phosphate buffer [7,12]. Therefore, many previously published studies on ginseng product quality control are limited to identifying neutral ginsenosides and ignoring ginsenoside Ro and malonyl ginsenosides.

Sample preparation is an important step that significantly affects the accuracy and repeatability of quantitative analysis. It has been reported that nonstandard sample pretreatment causes over 60% of analysis errors [29]. In a previous study in our laboratory, measurements of ginsenoside Ro and malonyl ginsenosides were evaluated by different extract solvents, such as methanol, ethanol, *n*-butanol, and water. The data indicated that the yields of ginsenoside Ro and malonyl ginsenosides in extraction solution with methanol were the highest and *n*-butanol were the lowest [8].

However, in most previous studies, the contents of ginsenoside Ro and malonyl ginsenosides from *Panax ginseng* were usually tested using HPLC with *n*-butanol as the extraction solvent, which negatively influenced the extraction efficacy of Ro and malonyl ginsenosides. Therefore, the content of ginsenoside Ro and malonyl ginsenosides could not be determined precisely.

Many studies have demonstrated variations in the content of total and individual ginsenosides in *P. ginseng* according to cultivation age, geographical origin, species, and various environmental conditions. Chung et al. [30] reported that total ginsenoside content in fresh *ginseng* was affected by cultivation age, whereas the effects of cultivation region were not significant for total ginsenoside level. Xiao et al. [17] showed that only the ginsenoside Rg_1_ and Re content increased with cultivation age of *P. ginseng*; the contents of Rb_1_, Rc, and Rd varied widely with cultivation ages from different cultivation locations. Another study suggested that the contents of Rb_1_, Rc, and Rb_2_ were affected by location; Re was affect by genotype; and Rg_1_ and Rd were affect by both location and genotype [18]. However, most previously published reports on variations in the content of total and individual ginsenosides in *P. ginseng* are limited to the determination of neutral ginsenosides. Very little work has been carried out on the accumulation characteristics of neutral and acidic ginsenosides of *P. ginseng* with different cultivation ages and harvest seasons.

In the present study, our results showed that the contents of ginsenoside Ro and malonyl ginsenosides in root increased during the two- to six-year-old stage. But the rate of increase was different. In particular, the dynamic changes in Ro content are significantly different than other saponins at different ages of *P. ginseng*. The Ro content in *P. ginseng* root was lowest in May of the second cultivation year and highest in September of the sixth cultivation year, ranging from 0.16 to 4.91 mg/g, with a difference about 30-fold (Table 1). Further, we found that the samples of 5 and 6-year-old *P. ginseng* had high Ro/Re ratio, whereas two- and three-year-old *P. ginseng* possessed a low Ro/Re ratio. The Ro/Re ratio presented a significantly large difference between cultivation ages of *P. ginseng*, which may cause differences in biological activity.

Interestingly, Wang et al. [20] showed that all samples of cultivated American ginseng grown in Wisconsin have the high Re/low Rg_1_ chemotype, whereas wild American ginseng samples are classified into two chemotypes based on the ratio Rg_1_/Re. Schlag and McIntosh [19] showed that chemotype did not vary by production type (wild versus cultivated American ginseng) and roots within a population rarely exhibited chemotypes different from the overall population chemotype. The chemotype variations in American ginseng populations are influenced more by genotype than environmental factors. Similarly, a significant variation in the saponin composition among Asian ginseng populations has been studied. According to the different expression patterns of core saponins, including Ra_1_/Ra_2_ and malonyl-Ra_1_/Ra_2_ isomers, Lee et al. [31] showed that the chromatographic patterns of core saponins could be approximately classified into three types, α, β and γ. The variations among ginsenosides play a critical role in discriminating between *P. ginseng* species. In our study, the results showed that the changes in the ratio of Re and Rg_1_ are clearly dependent on the harvest season of the *P. ginseng* root. May and June exhibit a low Rg_1_/Re ratio. July exhibits an intermediate ratio. August to October exhibit a high Rg_1_/Re ratio.

In general, ginseng roots harvested from the fifth to sixth year are considered to be the most valuable since they have the most mass and the highest amount of active components. But, it is likely that cheaper plant material and younger ginseng roots will appear in the marketplace [32]. Thus, a reliable quality control methodology to identify the age of *P. ginseng* is critical to prevent its adulteration in the market. However, it is difficult to apply traditional methods based on physical appearance to discriminating between ginseng products in slice or powder forms [9].

Recently, metabolomics research has been used to discriminate the cultivation age of ginseng products [13,24,25,26]. These metabolic approaches are usually combined with multivariate statistical analyses, which allow useful biological information to be extracted from complex metabolic data sets. However, the classification of samples with a massive volume of data is greatly challenging because numerous metabolites are analyzed by metabolome technique. Thus, it is important to improve data interpretability and reduce the sample size. This can be done by screening the influential metabolites from the data list that can be identified in samples. In this study, HCA and PCA were used to identify the cultivation age and season of *P. ginseng* using selected characteristic ginsenosides (ginsenosides Rg_1_, Re, and Ro). Our results showed that this method can be used to roughly discriminate the cultivation age and harvest season of *P. ginseng*. Kim et al. [25,33] reported the nontargeted metabolomics approach for age differentiation of *P. ginseng* using ultra-performance liquid chromatography quadrupole time-of-flight tandem mass spectrometry (UPLC-QTOFMS). The results showed that *P. ginseng* at different cultivation ages can be more precisely discriminated on the basis of selected metabolites. However, these studies only collected ginseng samples in fall. In our study, the samples of *P. ginseng* were collected from plants of different ages from May to October. It was easy to obtain similar chemical qualities among the samples with the close collection times. Therefore, it is difficult to completely distinguish different cultivation ages and collection months of *Panax ginseng*. Furthermore, many chemical components, including gluconic acid, glucuronic acid, proline, glucaric acid, mannose, myoinositol, panaxydol, and panaxynol, have also been found as key and relevant compounds with which to differentiate the age of ginseng samples [24]. However, whether these components have a better role in discrimination of cultivation ages and harvest seasons for *P. ginseng* root has not yet been explored. On this point, further experiments need to be performed.

## 4. Materials and Methods

### 4.1. Chemicals

Ginsenoside Rg_1_, Re, Rb_1_, Rc, Rb_2_ and Rd were purchased from College of Fundamental Medical, Jilin University (Changchun, China). Ginsenoside Ro and malonyl ginsenoside Rb_1_, Rc, Rb_2_, Rd were separated from fresh *Panax ginseng* in our group, and were identified using electrospray ionization mass spectrometry (ESI-MS), ^1^H-NMR and ^13^C-NMR spectroscopic data. All standards were at least 95% pure, as confirmed by HPLC. HPLC-grade acetonitrile and methanol were purchased from Merck (Darmstadt, Germany). Analytical grade methanol, ethanol and KH_2_PO_4_ were purchased from the Beijing Chemical Reagent Factory. Water was purified using a Milli-Q purification system (Millipore, Bedford, MA, USA). All solutions were filtered through a 0.45 μm hydrophilic polypropylene membrane before use.

### 4.2. Plant Materials

*P. ginseng* root samples used in this study were cultivated in Jingyu, Jilin Province, China, according to the ginseng GAP standard cultivation guidelines. Samples ranging from two to six years of age were collected in the same farm field from May to October in 2011. A total of 30 sample groups were collected (Table 2). For each group sample, five or six adjacent ginseng roots were collected at three to five different collecting points. The distance between the points was at least 3 meters. The voucher samples were authenticated by Dr. Sun Guang-zhi and deposited in the Institute of Agricultural Modernization at Jilin agricultural University (Jilin, China). The ginseng root was rinsed thoroughly with cold water, dried at 35 °C, powdered, and passed through a 40 mesh sieve.

### 4.3. Sample Preparation

One gram of root powder was put into a 50 mL conical flask. After adding 20 mL of 80% methanol solution (*v/v*), the flask was sonicated by KQ2200E Ultrasonic Cleaners (Kunshan Ultrasonic Instrument Co., Ltd., Kunshan, China) three times for 30 minutes. The extract was concentrated under a reduced pressure at 40 °C, and the residue was transferred into a 25 mL volumetric flask and diluted to the desired volume with methanol. The solutions were stored at 4 °C until HPLC analysis.

### 4.4. High Performance Liquid Chromatographic Analysis

An agilent 1100 series HPLC apparatus (Agilent Technologies, Santa Clara, CA, USA), equipped with quaternary gradient pump, vacuum degasser, autosampler and a UV detector was used for ginsenoside analysis. The standard ginsenoside mixture and the samples of extraction were dissolved in 25 mL of methanol and filtered through filters (0.45 µm (Millipore Corporation, Bedford, MA, USA)) for HPLC analysis. The separation was carried out on a 250 mm × 4.6 mm i.d., 5 μm, Cosmosil C18 column (Nacalai Tesque, Kyoto, Japan). For HPLC analysis, a 20 μL sample was injected into the column and eluted at 25 °C with a constant flow rate of 1.0 mL/min. The separation of ginsenosides was obtained by gradient elution (eluents was the mixture of acetonitrile (A) and 0.05 mol·L^−1^ KH_2_PO_4_ (B)). The process was according to the following profiles: 21% A at 0–22 min, 21–29% A at 22–30 min, 29% A at 30–50 min, 29–35% A at 50–60 min and 35–50% A at 60–70 min. The absorbance was measured at wavelength of 203 nm.

### 4.5. Multivariate Analysis

To identify and distinguish the cultivation ages and seasons, two multivariate statistical methods, the hierarchical cluster analysis (HCA) and the principal component analysis (PCA), were performed with SPSS version 20.0 software (SPSS, Inc., Chicago, IL, USA) and SIMCA-P 11.5 software (Umetrics, Umea, Sweden) respectively. HCA is a clustering method used to compare patterns of similarities and dissimilarities, which measures the distances among samples. A dendrogram represents the relationships among samples [34]. PCA was conducted to efficiently decrease the dimensions of the original data set. It explains the correlation among a large number of variables in terms of a smaller number of underlying factors without much information loss [35]. A dataset consisting of the detected ginsenoside levels of 30 *P. ginseng* samples was used to create the HCA dendrogram based on Within-groups linkage method. Furthermore, PCA was performed by applying a correlation matrix after by-step extracted pretreatment according to an Eigenvalue greater than 1.

### 4.6. Statistical Analysis

The statistical analysis was conducted based on a SPSS20.0 system (SPSS Inc., Chicago, IL, USA). Data are expressed as mean ± SEM. The ginsenoside contents of two groups were analyzed by independent samples *t*-test. Differences between the groups were analyzed using ANOVA. A difference of *p* < 0.05 was considered as being statistically significant.

## 5. Conclusions

To provide chemical characteristics for *Panax ginseng* quality control, dynamic changes in neutral and acidic ginsenosides in the *P. ginseng* root were investigated with different cultivation ages and different collection months using HPLC-UV. Our data showed that changes in the contents of ginsenoside Ro and malonyl ginsenosides were clearly dependent on the ginseng’s cultivation age, and the contents of ginsenosides Rg_1_ and Re were affected by the ginseng’s harvest season. Further, we found that the samples of five- and six-year-old *P. ginseng* had a high Ro/Re ratio, whereas two- and three-year-old *P. ginseng* possessed a low Ro/Re ratio. The *P. ginseng* samples from May to June exhibited a low Rg_1_/Re ratio, while the samples from August to October exhibited a high Rg_1_/Re ratio. Therefore, the Ro/Re and Rg_1_/Re ratios can be used as characteristic markers for the quality control of *P. ginseng*. In addition, we developed HPLC coupled with HCA and PCA to identify the cultivation age and harvest season of *P. ginseng* using characteristic ginsenosides (ginsenosides Rg_1_, Re, and Ro). Our results showed that this method can be used to discriminate the cultivation age and harvest season of *P. ginseng.*

## Figures and Tables

**Table 1:** Ginsenoside content and ratio in different cultivation age and month of *P. ginseng.*

Sample		Ginsenoside Content ^a^ (%)			Rg_1_/Re	Ro/Re
Month	Year	Rg_1_	Re	Ro		
	2	0.156	0.280	0.016	0.557	0.057
	3	0.251	0.316	0.072	0.794	0.228
May	4	0.270	0.320	0.169	0.844	0.528
	5	0.278	0.328	0.238	0.848	0.726
	6	0.301	0.330	0.331	0.912	1.003
	2	0.096	0.291	0.021	0.330	0.072
	3	0.228	0.296	0.101	0.770	0.341
June	4	0.253	0.301	0.215	0.841	0.714
	5	0.256	0.303	0.293	0.845	0.967
	6	0.276	0.316	0.364	0.873	1.152
	2	0.133	0.192	0.016	0.693	0.083
	3	0.228	0.205	0.145	1.112	0.707
July	4	0.226	0.212	0.217	1.066	1.034
	5	0.283	0.239	0.310	1.184	1.297
	6	0.308	0.306	0.421	1.007	1.376
	2	0.215	0.202	0.038	1.064	0.188
	3	0.244	0.209	0.142	1.167	0.679
August	4	0.245	0.211	0.253	1.161	1.199
	5	0.289	0.237	0.366	1.219	1.544
	6	0.358	0.278	0.486	1.288	1.748
	2	0.197	0.164	0.073	1.201	0.445
	3	0.209	0.168	0.103	1.244	0.613
September	4	0.256	0.196	0.240	1.306	1.224
	5	0.265	0.240	0.405	1.104	1.688
	6	0.331	0.251	0.491	1.319	1.956
	2	0.226	0.203	0.103	1.113	0.507
	3	0.310	0.227	0.207	1.366	0.912
October	4	0.312	0.236	0.286	1.322	1.212
	5	0.337	0.241	0.396	1.398	1.643
	6	0.343	0.257	0.446	1.335	1.735

^a^ Samples were tested in duplicate.

**Table 2:** *Panax ginseng* root sample collection information.

No.	Age (Year)	Date
1		12 May
2		15 June
3	Two	18 July
4		16 August
5		19 September
6		21 October
7		12 May
8		15 June
9	Three	18 July
10		16 August
11		19 September
12		21 October
13		12 May
14		15 June
15	Four	18 July
16		16 August
17		19 September
18		21 October
19		12 May
20		15 June
21	Five	18 July
22		16 August
23		19 September
24		21 October
25		12 May
26		15 June
27	Six	18 July
28		16 August
29		19 September
30		21 October
